# The Effect of Different Coverings on Total Body Score Development of Buried Carcasses

**DOI:** 10.21315/mjms2021.28.4.11

**Published:** 2021-08-26

**Authors:** Chee Hau Teo, Hiang Lian Hing, Noor Hazfalinda Hamzah, Sri Pawita Albakri Amir Hamzah

**Affiliations:** 1 Centre of Diagnostic, Therapeutic and Investigative Sciences, Faculty of Health Sciences, Universiti Kebangsaan Malaysia, Bangi, Selangor, Malaysia; 2 Forensic Science Analysis Centre, Department of Chemistry Malaysia, Petaling Jaya, Selangor, Malaysia

**Keywords:** decomposition, post-mortem changes, Total Body Score, burial condition, Thick Clothing, Plastic Wrapping

## Abstract

**Background:**

Examination of post-mortem changes is one of the ways to assess decomposition process on buried bodies. Nonetheless, studies on such assessment are still lacking, especially on the effects of body coverings by different materials in burial conditions. The aim of this research is to study the effect of different types of covering towards decomposition on buried rabbit carcasses by using Total Body Score (TBS) system.

**Methods:**

Twenty-seven rabbit carcasses were divided into: (i) No Clothing; (ii) Thick Clothing and (iii) Plastic Wrapping groups, and buried into individual shallow graves. One subject was exhumed from first to ninth post-burial week and assessed by using TBS system.

**Results and Discussion:**

There are significant differences among TBS between different coverings while controlling the time factor, *F* (2, 23) = 4.80, *P* < 0.05, partial *η*^2^ = 0.294. TBS score for Plastic Wrapping group is significantly lower than No Clothing group over times at *α* = 0.05, *P* < 0.05. The slightly delaying effect of thick clothing is caused by design of jacket, which allows arthropods access and microbial activity. Relatively strong delaying effect by plastic covering could be caused by impermeable property that caused accumulation of decompositional products and slow down the degradation.

**Conclusion:**

In conclusion, TBS system is a potential tool in describing rate of decomposition for buried cases in Malaysia.

## Introduction

The Greek word ‘taphe’ means grave or tomb, which reflects the focus of forensic taphonomy on how the dead body degrades into basic components in the decomposition process and the interactions between the decomposition process and surrounding ecology system variables (1–5). Potential information such as the presence of injuries, manner of death and post-mortem interval could be estimated by observing the pattern and extent of degradation that had occurred, which then helps in criminal case investigation (6, 7). Decomposition process itself is subject to the influence of numerous intrinsic and extrinsic factors, including age, size of body, medical condition before death, trauma and injuries, ambient temperature, moisture, covering, burial, accessibility of arthropod, scavenger animal activity and so on (8–11). However, the effect of each factor varies across different geographical conditions and seasons (2, 12, 13).

Under normal circumstances, buried bodies are generally believed to decompose at a slower rate as compared to the bodies on ground surface, which are exposed to much more decomposer factors (4, 7, 14–17). Other than lower underground temperature, the restricted arthropod access in burial condition is also attributed to this phenomenon (4, 6, 7, 18, 19). The soil layer blocks significant amount of heat from sunshine and the increased depth of grave further reduces the temperature and delays the rate of decomposition. The faunal succession is also different between bodies exposed on the ground surface and buried bodies in the grave and huge larvae mass is rarely reported in latter condition (14, 15, 20, 21). Besides that, the rate of decomposition in grave could also be affected by other factors, such as the presence of covering, coffin, physical and chemical properties of soil (22–25).

Covering plays a significant role in influencing the post-mortem changes and estimation of post-mortem interval. In general, clothing will delay the arrival of Dipteran insects on the corpse and slow down the rate of decomposition and subsequent post-mortem changes (6, 20, 26). However, previous studies had discovered that light or thin clothing still allows high degree of exposure of body parts hence has no delaying effect on the Dipteran infestation (27, 28). Fabrics made of synthetic materials are found to have higher resistance against microbial degradation as compared to natural materials (22, 25). When combined with moisture released from decomposition process and surrounding, different types of fabric will lead to other post-mortem changes deviations, including mummification and formation of adipocere due to the differences in moisture adsorption property (18, 22, 25, 26).

Conventional assessments for decomposed bodies by forensic pathologists are mainly based on physical observation of post-mortem changes and stages of soft tissue degradation that had occurred. Estimation of post-mortem interval in such cases largely depends on the experience of the forensic experts with the support of extensive information and aid of relevant researches (9, 10, 29). The qualitative description of remains on post-mortem changes in decomposition process could be very subjective and findings between cases often could not be compared between each other due to different conditions in various circumstances (5, 30). Furthermore, the rate of decomposition process may not be the same for different body parts; hence post-mortem changes from multiple stages of degradation can be found on the same corpse simultaneously (13, 31, 32).

Since post-mortem changes often occur in a predictive sequence, the application of numerical system in scoring during the assessment of the extent of decomposition is possible. Total Body Score (TBS) system is a relatively new system developed by Megyesi et al. (33) that allows quantitative assessment on decomposition process based on three main body parts, which are: (i) head and neck; (ii) body trunk; and (iii) hands and legs. TBS provides scoring method starting from fresh stage until dry remains stage and corresponds to certain post-mortem changes that could be observed on specific to particular body parts (2, 13, 33, 34).

The main purpose of this study is to examine the effect of different types of covering onto the decomposition process on rabbit carcasses buried in shallow graves and the co-relationship and differences between these. TBS system shall be used for assessment of the extent of decomposition process of the buried subjects. Currently, there are very few decomposition studies conducted which focus on buried cases and different types of coverings in Malaysian local condition.

## Methods

Due to the absence of specialised research facility that allows the utilisation of human corpse in decomposition studies, rabbit (*Oryctolagus cuniculus*) was used as subject in this study due to its much stable source and more cost-effective as compared to other alternative species. Previous studies revealed that the decomposition process of buried subjects throughout six post-burial weeks with two weeks exhumation interval was not adequate for the subjects to reach skeletonised stage (28). Therefore, with increased sample size and more frequent observation intervals in this study, 27 adult rabbit carcasses weighing from 2 kg to 4 kg were used in this study, approved by the Animal Ethics Committee of Universiti Kebangsaan Malaysia. All rabbits were obtained from Animal House, Faculty of Science and Technology, Universiti Kebangsaan Malaysia and bred under controlled condition without illness before euthanasia. The subjects were randomly separated into three covering groups: (i) No Clothing (as the control group); (ii) Thick Clothing; and (iii) Plastic Wrapping. The Thick Clothing setting includes long sleeve shirts, long pants, socks and zipped jackets, all of which are made of cotton. For the Plastic Wrapping group, each rabbit carcass was put inside a black polyethylene rubbish bag with air void removed before it was tied with a cable tight.

All carcasses were enclosed in separate wire mesh cages and buried in individual 1 ft depth graves with a distance of at least 3 m apart to avoid any interference from one another while ensuring identical microclimate among the graves (11, 15). All shallow graves were located at the Forensic Science Programme Simulation Site, Universiti Kebangsaan Malaysia, Bangi Campus. Wire mesh cages were used for protection against scavenger animal activity and digging action during exhumation while mimicking the original exposure of carcasses to soil in burial condition. Insect activities were recorded if present (35).

One carcass from each group was exhumed weekly from first until ninth post-burial week for decomposition process assessment. The burial period was designed to be as close as possible from early September to mid December 2013. Post-mortem changes that occurred were examined and scored using the TBS system. Although TBS system was previously developed based on human corpses, this system was proven to yield satisfactory outcomes in previous decomposition studies by using rabbit carcasses as alternatives for human corpses (14, 31, 36). In this study, hands and legs on human corpse were demonstrated by fore and hindlimbs on rabbit carcass for the TBS system assessment. As shown in Tables 1, 2 and 3, the rate of decomposition on different body parts could be scored independently in TBS system based on observation of post-mortem changes, thus overcoming the aforementioned phenomenon of various stages of decomposition on the same body. The total mark from three body parts is the TBS, reflecting the current extent of the overall decomposition process on the buried subjects. The TBS for all carcasses were recorded as soon as possible after exhumation and the development of the score in all groups was then plotted against the number of post-burial weeks. One-way analysis of covariance (ANCOVA) was used to compare the TBS recorded between three different types of covering while the number of post-burial weeks was included as a covariate to partial out its effect.

## Results and Discussion

Generally, the development of the decomposition process and the post-mortem changes observed were aligned with the scoring table used in TBS system. The TBS recorded for all three covering groups were shown in Table 4. Until the last exhumation at the ninth post-burial week, none of the subjects showed total dry bones for all body regions. The body trunk region for No Clothing and Thick Clothing group subjects entered in skeletonisation stage while the Plastic Wrapping group subjects were observed as still in advanced decomposition condition.

**Figure 1:**
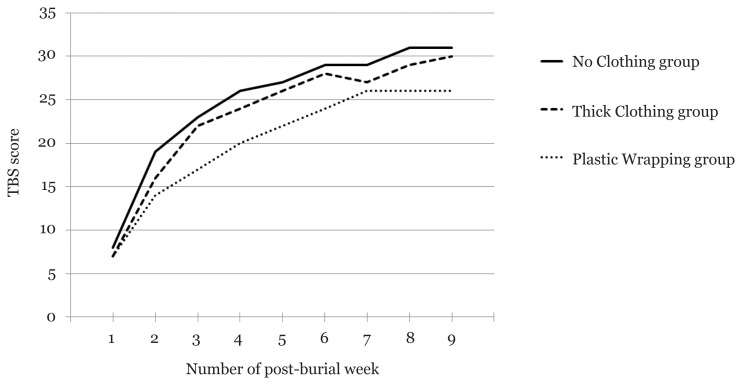
Development of TBS against the number of post-burial weeks Note: TBS of each subject is the sum of score from three body parts

The TBS of all three covering groups were almost alike in the first two post-burial weeks initial period. A noticeable increase in TBS of No Clothing group was recorded on the third post-burial week and closely followed by Thick Clothing group on the fourth post-burial week exhumations. The development of TBS for Thick Clothing group closely followed that of No Clothing group until the late phase of the study. However, the increase of TBS in Plastic Wrapping group was found to be slower than the other two groups. The development of the TBS in all three covering groups was shown in Figure 1.

Statistical analysis one-way ANCOVA results showed that the TBS for all three different covering groups were normally distributed and homogeneity of regression slopes and homogeneity of variances were supported by a non-significant covariate interaction, *F* (2, 21) = 0.05, *P* = 0.949 and a non-significant Levene’s test, *F* (2, 24) = 0.47, *P* = 0.631, respectively. The number of post-burial week is significantly related to the TBS, *F* (1, 23) = 98.38, *P* < 0.001; and there were significant differences between the type of covering factor after controlling post-burial week factor, *F* (2, 23) = 4.80, *P* < 0.05, partial *η*^2^ = 0.294. Post-hoc testing revealed that the score for Plastic Wrapping group is significantly lower than that of No Clothing over times at α = 0.05, (Sig = 0.017). There was, however, no significant statistical difference between the remaining pairwise comparison at α = 0.05.

The head and neck region for all groups entered early decomposition stage during the first post-burial week exhumation and reached advanced decomposition in the second post-burial week for No Clothing and Plastic Wrapping groups; and in the third week for Thick Clothing group. This region already entered skeletonisation stage on the fourth post-burial week, showing more than 50% degree of bone exposure in No Clothing and Thick Clothing group. The carcasses in Plastic Wrapping group, on the other hand, only recorded skeletonised stage score on the eighth post-burial week. Only subjects in No Clothing group recorded full score in skeletonisation stage on the head and neck region on the ninth week post-burial exhumation.

The body trunk region was noted to decay slower than the other two body parts. This agrees with Brown and Peckmann (37) findings and could be due to the extensive amount of tissue in this region. Observations showed that the body trunk region of all groups was in early decomposition during the first exhumation. Body trunk part of No Clothing group subjects was the earliest to enter advanced decay stage on the second post-burial week as compared to Thick Clothing and Plastic Wrapping group subjects that reached the same stage during the fourth and fifth post-burial weeks, respectively. The TBS for the body trunk region for No Clothing and Thick Clothing groups’ carcasses reached skeletonisation stage during the last exhumation of the study while the body trunk of Plastic Wrapping group carcass was still in advanced decomposition condition. On the other hand, the fore and hindlimbs region was found to decay at the fastest rate. The limbs region in all covering groups entered skeletonisation stage as early as the third week for No Clothing group and the fourth week for Thick Clothing and Plastic Wrapping groups. The limbs region of all covering groups recorded full TBS for skeletonised stage on the sixth post-burial week onwards.

This study found that TBS system has significant potential to become an alternative supplementary approach for post-mortem changes assessment in burial subjects. TBS system provides numerical data for evaluation of decomposition when different body parts of carcass may decay at different rates, as mentioned by Adlam and Simmons (31). Previous studies proved the correlation between TBS scoring and accumulated degree days in cases with various post-mortem changes conditions, including buried and submerged cases (14, 38, 39). However, there is a dearth of previous studies which apply TBS system assessment on the decomposition process of buried bodies in relation to the presence of clothing. The outcome of this study indicated that this system showed positive prospects in establishing statistical comparisons between different cases more objectively.

Previous studies Teo et al. (28) had used rabbit carcasses with similar clothing settings and burial condition, but the recorded TBS of the subjects of the same burial period in this study were generally lower. This could be explained by the location of shallow graves in the previous research which has high sun exposure and higher soil temperature and thus higher rate of decomposition as compared to shallow graves in this study which were all located in shady areas (14, 40). We also noticed that the rapid development of TBS in this study further proved that the rate of decomposition in Malaysia is much faster as compared to similar studies in temperate climate regions, strengthening findings from previous local studies (28, 35). Bachmann and Simmons (14) found that TBS scoring for No Clothing buried rabbit carcass required 50 days to reach the TBS ranges from 20 to 26, while the subjects of similar condition in this study only need three weeks (21 days) to reach the same range of TBS. Other than the warmer climate in Malaysia, this could also be explained by the differences in soil, geographical conditions with more active faunal succession and microbiological population.

Thick cotton clothing was found to possess a slight delaying effect on the overall decomposition process compared to the No Clothing group subjects although it was not statistically shown. This correlates with the general opinion that thick clothing will delay the detection of dead bodies by arthropods and further delay their accessibility. However, the development of TBS in Thick Clothing group in this study was found to follow the No Clothing group scoring closely. This could be explained by the design of jacket and shirt in this study, which still exposes a large area of the face and neck region of the carcass compared to the complete enclosure in the Plastic Wrapping setting. Although pre-burial arthropod activity exposure was avoided, the exposed face and neck still serve as potential oviposition sites for the normal burial faunal succession process on buried bodies. Natural orifices present at the head and neck region could trigger the onset of larval infestation and further lead to faster decomposition in this body region (28, 41). At the same time, we recorded a small amount of arthropod pupae between the jacket and long sleeves of clothes among the subjects. Previous studies stated that clothing may function as a protective layer for larvae from hazards and prolongs arthropod activity while serving as an ideal site for pupation (24, 27, 42–44). Some studies also noted that cotton clothing may induce desiccation of tissue (19, 22, 25). However, this was not observed in this study due to the condition of burial where airflow is very limited in the grave and the soil which does not enhance dispersion of moisture away from the carcasses. This is supported by previous studies that mentioned the effect of clothing in retaining moisture and prolongs the period of advanced decay stage of decomposition (43, 45).

The delay effect of plastic wrapping in decomposition is clearly shown from the slow development of the TBS and statistical result in this study and supported by previous studies (25). As aforementioned, there was a significant difference between the TBS recorded for subjects in Plastic Wrapping group and No Clothing group. However, the extent of the delaying effect of Plastic Wrapping and Thick Clothing could not be quantified statistically during this initial phase of research. Of interest, there was no significant amount of adipocere spotted in the Plastic Wrapping group although some previous studies proposed that the formation of adipocere could be found on bodies found in water, clothed or plastic settings (11, 25, 29, 37, 45). This could be due to the impermeable nature of plastic that had caused the retention of decompositional fluid around the carcass and the accumulated decompositional products within the plastic have an adverse effect on the decomposition process (11, 46, 47). Troutman et al. (48) noticed that adipocere could mostly be found on deep carcass due to the accumulation of liquefied fat compounds from the mass grave. However, the grave setting in this study is only 1 ft deep and the carcasses were buried individually.

It is recommended that an increase of the sample size with a more extended burial period since different covering test groups did not reach the full skeletonisation TBS by the end of the last exhumation procedure. Other types of clothing including silk, wool and polyester could also be studied. In addition to the study scope, there is a wide range of fabrics and materials available in the market; each type of covering has different aforementioned moisture retention and adsorption properties that demonstrate different effects on tissue degradation. Therefore, further studies that focus on a variety of fabrics and materials with increased sample size could help in understanding the influence and the degree of differences between types of covering on the rate of decomposition in buried body cases in the Malaysian forensic context.

## Conclusion

This study showed the feasibility of TBS system in assessing the post-mortem changes of buried carcasses under the influence of Thick Clothing and Plastic Wrapping conditions. TBS system could function as a supporting tool and provide statistical basis for experts in examining the extent of decomposition and estimating post-mortem interval of buried corpses in actual cases. Limb region on buried rabbit carcasses decomposed at the fastest rate, followed by the head and neck region and lastly, the body trunk region due to the significant difference in tissue mass volume. The development of TBS in No Clothing setting was the fastest, followed by Thick Clothing and Plastic Wrapping conditions. There are significant differences among the TBS of rabbit carcasses buried between different types of covering within a similar time frame of burial in shallow graves. The delaying effect of Thick Clothing was not significantly observed as it still allows access of arthropod at exposed face and neck region as compared to Plastic Wrapping, which completely covers the whole carcass. The slowest rate of TBS development in the Plastic Wrapping group could be explained by the impermeable property of plastic which caused the accumulation of decompositional products from the degradation process. This alters the internal environment within the plastic into an unfavourable setting for the decomposition process. The decomposition process in Malaysia occurs at a faster rate as compared to temperate climate regions.

## Figures and Tables

**Table 1:** TBS for the region (i) head and neck

Stage of decomposition	Score	Observation of post-mortem change
Fresh	1	Fresh, no discolouration

Early decomposition	2	Pink-white appearance with skin slippage and some hair loss
	3	Gray to green discolouration: some flesh still relatively fresh
	4	Discolouration and/or brownish shades particularly at edges, drying of nose, ears and lips
	5	Purging of decomposition fluids out of eyes, ears, nose, mouth, some bloating of neck and face may be present
	6	Brown to black discolouration of flesh

Advanced decomposition	7	Caving in of the flesh and tissues of eyes and throat
	8	Moist decomposition with bone exposure < 50% of the area being scored
	9	Mummification with bone exposure < 50% of the area being scored

Skeletonisation	10	Bone exposure > 50% of the area being scored with greasy substances and decomposed tissue
	11	Bone exposure > 50% of the area being scored with desiccated or mummified tissue
	12	Bone largely dry but remaining some grease
	13	Dry bone

Source: Dautartas (22)

**Table 2:** TBS for the region (ii) body trunk

Stage of decomposition	Score	Observation of post-mortem change
Fresh	1	Fresh, no discolouration

Early decomposition	2	Pink-white appearance with skin slippage and marbling present
	3	Gray to green discolouration: some flesh still relatively fresh
	4	Bloating with green discolouration and purging of decomposition fluid
	5	Post bloating following release of the abdominal gases, with discolouration changing from green to black

Advanced decomposition	6	Decomposition of tissue producing sagging of flesh; caving in of the abdominal cavity
	7	Moist decomposition with bone exposure < 50% of the area being scored
	8	Mummification with bone exposure < 50% of the area being scored

Skeletonisation	9	Bones with decomposed tissue, sometimes with body fluids and grease still present
	10	Bones with desiccated or mummified tissue with bone exposure < 50% of the area being scored.
	11	Bone largely dry but remaining some grease
	12	Dry bone

Source: Dautartas (22)

**Table 3:** TBS for the region (iii) fore and hindlimbs

Stage of decomposition	Score	Observation of post-mortem change
Fresh	1	Fresh, no discolouration

Early decomposition	2	Pink-white appearance with skin slippage of forelimbs and/or hindlimbs
	3	Gray to green discolouration: some flesh still relatively fresh
	4	Discolouration and/or brownish shades particularly at edges, drying of fingers, toes and other projecting extremities
	5	Brown to black discolouration: skin having a leathery appearance

Advanced decomposition	6	Moist decomposition with bone exposure < 50% of the area being scored
	7	Mummification with bone exposure < 50% of the area being scored

Skeletonisation	8	Bone exposure < 50% of the area being scored, some decomposition tissue and body fluids remaining
	9	Bone largely dry but remaining some grease
	10	Dry bone

Source: Dautartas (22)

**Table 4:** TBS scores recorded for No Clothing, Thick Clothing and Plastic Wrapping groups

Number of post-burial weak	No Clothing	Thick Clothing	Plastic Wrapping
1	8	7	7
2	19	16	14
3	23	22	17
4	26	24	20
5	27	26	22
6	29	28	24
7	29	27	26
8	31	29	26
9	31	30	26
